# Epitranscriptomics, immunometabolism, and NETosis: unlocking novel mechanisms of traditional Chinese medicine in COPD

**DOI:** 10.3389/fimmu.2026.1904338

**Published:** 2026-08-04

**Authors:** Hui-Hui Chen, Feng-Xian Ni, Dong-Hui Huang, Ze-Bo Jiang

**Affiliations:** 1 Zhuhai Hospital of Integrated Traditional Chinese and Western Medicine, Zhuhai, Guangdong, China; 2 Zhuhai Hospital Affiliated to Faculty of Chinese Medicine, Macau University of Science and Technology, Zhuhai, Guangdong, China

**Keywords:** COPD, immunometabolism, inflammation resolution, m6A methylation, Malt1, METTL16, NEtosis, traditional Chinese medicine

## Abstract

Chronic obstructive pulmonary disease (COPD) is driven by self-perpetuating inflammation that resists conventional therapies. Recent evidence reveals three interconnected pathogenic layers: immunometabolic reprogramming, m^6^A epitranscriptomic dysregulation, and excessive NETosis with defective resolution. Pathogenic immune cells shift toward aerobic glycolysis and impaired fatty acid oxidation, sustaining pro-inflammatory phenotypes and corticosteroid resistance. m^6^A modifications may control the stability of key transcripts such as MALT1, IL-17, and PADI4, as suggested by emerging evidence. A recent rat COPD model study reported that the Shenqi Tiaoshen Formula reduced METTL16-mediated m^6^A methylation of MALT1, though human replication is lacking. Concurrently, neutrophil extracellular traps (NETs) damage lung tissue and expose autoantigens, accompanied by failed resolution. Traditional Chinese Medicine (TCM)—including Shenqi Tiaoshen Formula, baicalin, berberine, astragaloside IV, and curcumin—can reprogram metabolism via AMPK, modulate m^6^A writers/erasers, inhibit NETosis, and upregulate SPMs in preclinical models. However, most evidence derives from non-COPD models or uses supraphysiological concentrations; the translational gap to human COPD remains substantial. We discuss the bidirectional metabolism-m^6^A crosstalk as a theoretical framework requiring experimental validation, and outline single-cell m^6^A sequencing, spatial metabolomics, and CRISPR screening as tools to accelerate precision TCM for COPD.

## 1 Introduction

Chronic obstructive pulmonary disease (COPD) remains a leading cause of death worldwide. Current treatments provide only symptomatic relief without reversing the underlying immune pathology (1, 2). The failure of conventional therapies stems from the complex, self-reinforcing nature of COPD inflammation (3, 4). Bronchodilators and inhaled corticosteroids, the mainstays of current pharmacotherapy, alleviate symptoms and reduce exacerbation frequency but do not halt disease progression or reverse established structural damage such as emphysema and airway remodeling. This therapeutic plateau reflects a fundamental gap in our understanding of the molecular circuits that sustain chronic inflammation long after smoking cessation, the primary risk factor in most cases (5–7).

Over the past decade, three previously distinct areas have converged to reveal a self-reinforcing pathogenic network. Immunometabolism describes how metabolic pathways dictate immune cell function (8–12). Epitranscriptomics—specifically N^6^-methyladenosine (m^6^A) mRNA modification—has emerged as a central post-transcriptional regulator, whose enzymes are sensitive to cellular metabolites, linking metabolism to RNA methylation (13–16). Finally, NETosis causes tissue damage, promotes autoimmunity, and is exacerbated by defective inflammation resolution due to low specialized pro-resolving mediators (SPMs) (17, 18).

Traditional Chinese Medicine (TCM), with its multi-component, multi-target nature, is uniquely suited to intervene in such complex networks (19–21). A landmark study in a rat model showed that Shenqi Tiaoshen Formula reduces METTL16-mediated m^6^A methylation of *MALT1* mRNA, suppressing airway inflammation—the only direct mechanistic demonstration of TCM-induced epitranscriptomic modulation in COPD, though awaiting human replication (22). Other compounds (baicalin, berberine, astragaloside IV, curcumin) modulate metabolic sensors, inhibit NETosis, and upregulate SPMs, but mostly in non-COPD contexts or at supraphysiological concentrations (23, 24).

This review integrates these dimensions into a unified narrative. We describe immunometabolic reprogramming and TCM interventions, discuss m^6^A dysregulation and its bidirectional crosstalk with metabolism, examine NETosis and resolution failure, and synthesize a network model with emerging technologies for precision TCM.

## 2 Immunometabolic reprogramming in COPD and TCM intervention

### 2.1 Metabolic phenotypes of key immune populations in the COPD lung

In healthy lungs, alveolar macrophages maintain tissue homeostasis through oxidative metabolism characterized by fatty acid oxidation (FAO) and oxidative phosphorylation (OXPHOS) (25). In COPD, macrophages shift to aerobic glycolysis—marked by upregulated GLUT1, HK2, and PKM2, elevated lactate, and reduced mitochondrial respiration—driven by mTORC1–HIF-1α signaling (26). Downregulated CPT1A and impaired FAO sustain M1 polarization (27), increasing ROS, inflammasome activation, impaired efferocytosis, and protease secretion (28–30). This reprogramming is not fully reversible upon smoking cessation (26).

Neutrophils are inherently glycolytic, relying on glycolysis and the pentose phosphate pathway (PPP) for rapid ATP and NADPH (31–33). In COPD, neutrophils exhibit exaggerated glycolytic flux, fueling NOX2-dependent respiratory bursts and NETosis (32, 34, 35). Glycolytic metabolites stabilize Mcl-1, delaying apoptosis and prolonging tissue residence (36, 37), while impaired macrophage clearance exacerbates damage (15, 38, 39).

CD4^+^ T cell subsets adopt distinct metabolic dependencies. Th17 cells engage aerobic glycolysis and glutaminolysis, supporting IL-17 proliferation (40–43), with upregulated mTORC1 and HIF-1α in COPD (44–46). Regulatory T cells depend on FAO and OXPHOS for stability (47). COPD lungs show Treg defects—reduced CPT1A, decreased mitochondrial spare respiratory capacity, and diminished Sirtuin/PGC-1α signaling (48–52). The resulting Th17/Treg imbalance drives neutrophilic inflammation and corticosteroid insensitivity (53). ILC2s rely on FAO, while ILC3s are glycolytic; during exacerbations, shifts favoring ILC3 expansion contribute to neutrophil recruitment (53–55).

### 2.2 Key metabolic regulators and their dysregulation in COPD

The mTORC1-HIF-1α axis promotes glycolysis and pro-inflammatory gene expression (56, 57). In COPD, chronic oxidative stress and persistent pattern recognition receptor signaling maintain pathological mTORC1 activation, reinforcing glycolytic dominance across macrophages, Th17 cells, and neutrophils (58). Pharmacological inhibition of mTORC1 with rapamycin or its analogs attenuates cigarette smoke-induced inflammation in preclinical models, supporting the therapeutic relevance of this axis (59, 60). AMP-activated protein kinase (AMPK), the principal energy sensor, promotes FAO and antagonizes mTORC1 (61). AMPK activation shifts metabolism toward OXPHOS, supporting anti-inflammatory phenotypes (62). Reduced AMPK activity in COPD favors succinate accumulation, which inhibits m^6^A erasers FTO and ALKBH5 (63, 64). Agents that activate AMPK, such as metformin and certain TCM compounds, have shown promise in COPD models and are being evaluated in clinical trials (65–68). PPARγ support M2 polarization and Treg stability (69). Downregulation of PPARγ in COPD contributes to the loss of reparative phenotypes and impaired resolution (69). PPARγ agonists, including astragaloside IV, demonstrate anti-inflammatory effects (70, 71).

### 2.3 Metabolic modulation of effector functions and clinical consequences

NET formation requires rapid glycolytic flux and PPP-derived NADPH for ROS generation (72). Hyperglycolytic neutrophils in COPD are predisposed to exaggerated NETosis (34). Interventions shifting metabolism away from glycolysis, such as AMPK activation or glucose restriction, reduce NET formation (73, 74).

Metabolism modulates corticosteroid responsiveness. High glucose and glycolytic signaling activate p38 MAPK, phosphorylating the glucocorticoid receptor and impairing its function (75, 76, 77). This explains poor steroid response in neutrophilic COPD endotypes (78). Metabolic normalization may resensitize cells to steroids (78). Metabolic stressors, such as succinate accumulation and one-carbon metabolism dysregulation, influence epigenetic landscapes and autoantigen generation, while diminished SPM synthesis due to impaired PPARγ signaling undermines resolution (79).

### 2.4 TCM interventions that recalibrate immunometabolism

Preclinical evidence demonstrates that TCM formulas and compounds can shift immune metabolism toward reparative states (80), though most mechanistic data derive from non-COPD models and require validation in COPD-relevant systems. The Shenqi Tiaoshen Formula has been shown to ameliorate inflammatory phenotypes and promote M2 polarization in cigarette smoke and LPS-exposed rat models (81, 82). Metabolomic analyses revealed reduced lactate and pyruvate levels in bronchoalveolar lavage fluid, increased expression of fatty acid oxidation genes such as *CPT1A* and *ACADM*, and enhanced mitochondrial function. Importantly, this formula also downregulates METTL16 and reduces m^6^A methylation of *MALT1* mRNA, thereby linking metabolic reprogramming to epitranscriptomic regulation (83). This finding, while mechanistically intriguing, comes from a single study in a rat model; it has not been replicated in human primary cells or clinical samples, and its generalizability to human COPD remains to be established.

Among classic herbal formulas, the Bufei Yishen Formula has been reported to upregulate PPARγ, restore FAO markers, and reduce lactate and pyruvate levels, correlating with improved lung mechanics and reduced inflammation. Network pharmacology analyses identified AMPK, PPARγ, and PGC-1α as key targets, and experimental validation confirmed activation of this axis. Berberine, an AMPK activator, suppresses glycolysis, fosters FAO, reduces Th17 differentiation (EC_50_ ∼1-5 µM in murine T cells (84, 85); human data limited), and restores Treg numbers (86–88). Preclinical studies suggest enhanced steroid responsiveness (89), but clinical efficacy data in COPD are lacking. Astragaloside IV, a natural PPARγ agonist with an IC_50_ of approximately 10 µM in competitive binding assays using recombinant human PPARγ (90). Additionally, astragaloside IV activates the SIRT1/PGC-1α pathway through NAD^+^-dependent mechanisms, enhancing mitochondrial biogenesis (91). It improves macrophage phagocytosis and inhibits HIF-1α (92–95). Recent evidence indicates that astragaloside IV can directly regulate key elements of the m6A machinery, including the modulation of METTL3 expression in certain pathological contexts (96). Baicalin inhibits glycolytic enzymes HK2 and PKM2 (IC_50_ ∼15-30 µM in cancer cell lines (97)), modulates ILC2/ILC3 balance, and reduces neutrophil NETosis (97, 98). m^6^A-modulating effects reported in lung cancer models (not COPD) (97, 99). Curcumin inhibit mTORC1 (100) and activate AMPK through multiple pathways, potentially involving LKB1 (101). It suppresses Th17 cell differentiation while promoting Treg differentiation (102, 103), with m^6^A effects in colitis models (104). Resveratrol activate SIRT1 (105), inhibits HIF-1α (106)and reduces inflammasome activation (107).

**Figure 1:**
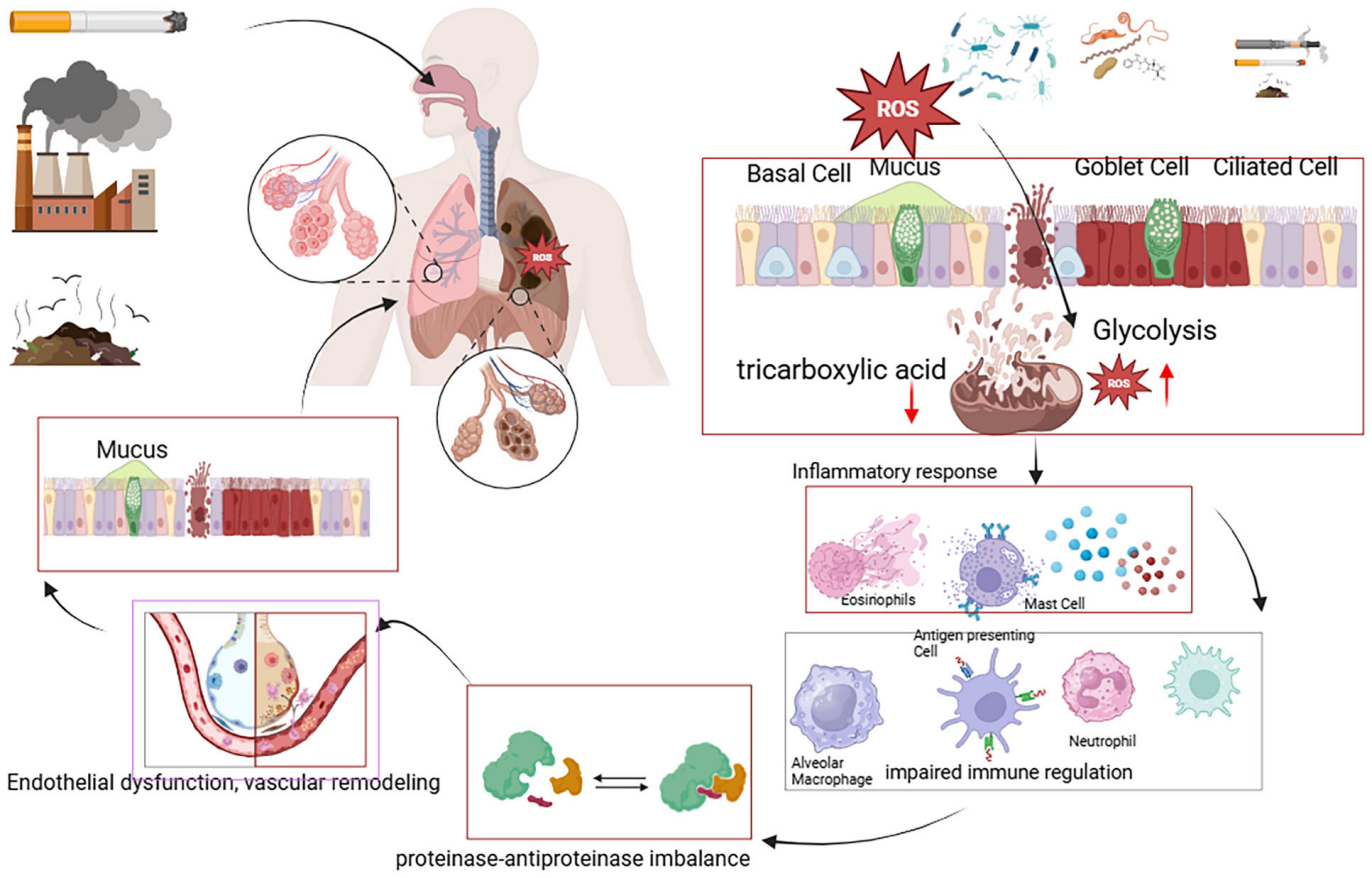
Pathogenic cascade of chronic obstructive pulmonary disease (COPD) induced by environmental insults and metabolic reprogramming. Chronic environmental insults, including cigarette smoke, air pollutants, and biomass exposure, drive pathological changes in the lungs. These triggers induce reactive oxygen species (ROS) production, which disrupts cellular metabolism in airway epithelial cells: the tricarboxylic acid (TCA) cycle is suppressed, while glycolysis is enhanced. This metabolic shift initiates a cascade of events: activation of inflammatory responses involving eosinophils, mast cells, and impaired immune regulation by alveolar macrophages, antigen-presenting cells, and neutrophils. The sustained inflammatory state leads to mucus hypersecretion, goblet cell hyperplasia, and ciliary dysfunction in the airway epithelium. Concurrently, the imbalance between proteinases and antiproteinases promotes extracellular matrix degradation, ultimately resulting in endothelial dysfunction and vascular remodeling. These interconnected processes perpetuate a self-amplifying cycle of tissue damage and progressive lung destruction in COPD.

The concentrations above are *in vitro* EC_50_ values, generally higher than achievable systemic levels (see Section 2.6), raising questions about clinical relevance. These TCM interventions converge on enhanced FAO and OXPHOS with suppressed glycolysis, but direct validation in human COPD at relevant doses is urgently needed (**Figure 1**).

### 2.5 Integrative mechanisms and clinical translation

The interplay between cellular metabolism and m^6^A RNA methylation is bidirectional and will be discussed in detail in Section 3. Briefly, TCM compounds that lower glycolysis, reduce succinate accumulation, or modulate one-carbon metabolism have the potential to restore m^6^A homeostasis and reverse pathogenic transcript stabilization (108). By shifting neutrophil and macrophage metabolic states away from glycolysis and toward fatty acid oxidation and oxidative phosphorylation, TCM agents decrease NET formation and enhance macrophage efferocytosis (109). Additionally, by upregulating PPARγ and 15-lipoxygenase, these agents promote SPM biosynthesis, re-establishing active resolution and tissue repair (110).

Early clinical data are promising but limited. Systematic reviews of Qingjin Huatan decoction and Yupingfeng San suggest benefits, but lack quality is low (111). No large-scale RCTs of TCM in COPD have been published. A pilot berberine study in hyperglycemia improved metabolic parameters, but was not conducted in COPD patients (112). Translation faces challenges: off-target systemic effects, need for pulmonary selectivity, long-term safety, and biomarker-driven stratification (sputum lactate, succinate, SAM/SAH, CPT1A, NET burden, m^6^A status). Causal hierarchy and temporal dynamics remain unclear.

### 2.6 Pharmacokinetic considerations and bioavailability challenges

Many TCM compounds studied *in vitro* at concentrations not achievable *in vivo*. Berberine has extremely poor oral bioavailability (<1%) (113); effective AMPK activation *in vitro* (1-5 µM) exceeds typical plasma levels (114). Baicalin also has poor oral bioavailability due to glucuronidation (115), its aglycone baicalein is better absorbed (116). Astragaloside IV has moderate oral bioavailability (approximately 3.7-7.4%) (117), with lung tissue distribution favorable (118). Curcumin has exceptionally poor oral bioavailability due to rapid metabolism, poor aqueous solubility, and rapid systemic clearance (119). Resveratrol has moderate oral bioavailability but rapid metabolism (120). Lung-targeted delivery systems and PK/PD modeling are critical for translation.

### 2.7 Drug-drug interactions and safety considerations

TCM compounds may interact with standard COPD therapies via CYP enzymes and transporters. Berberine inhibits CYP2D6, CYP3A4, and CYP2C9 (121); baicalein inhibits CYP3A4 and CYP2C9 (122); curcumin inhibits CYP3A4 (123); resveratrol inhibits CYP3A4 and CYP1A1 (124). Berberine is both a substrate and an inhibitor of P-glycoprotein (ABCB1) (125); baicalin and its aglycone baicalein have been reported to inhibit organic anion transporting polypeptides (OATPs), including OATP1B3 and OATP2B1 (126). Preclinical data suggest berberine and astragaloside IV may enhance steroid responses (89, 127, 128). Practical strategies include dose titration, staggered administration, therapeutic drug monitoring, and systematic adverse event monitoring in trials.

## 3 m^6^A RNA methylation in COPD and TCM intervention

N^6^-methyladenosine is the most prevalent internal modification, regulating stability, splicing, localization, and translation (129). Although direct evidence for epitranscriptomic dysregulation in COPD is nascent, emerging data implicate m^6^A as a driver and therapeutic target. TCM formulas influencing both metabolism and m^6^A provide a promising avenue (68, 130–132).

### 3.1 Molecular machinery of m^6^A and its immune functions

m^6^A is deposited co-transcriptionally by a multisubunit methyltransferase complex whose catalytic core comprises METTL3 and METTL14 (133). Accessory factors including WTAP, VIRMA, RBM15/15B, ZC3H13 confer substrate selectivity, facilitating methylation at consensus RRACH motifs preferentially near stop codons and in 3′UTRs (134, 135). METTL16 is a distinct writer that methylates a separate repertoire of RNAs, notably *MAT2A*, the gene encoding methionine adenosyltransferase, which catalyzes SAM synthesis, thereby connecting m^6^A deposition to cellular methylation potential (136–138). m^6^A marks are removed by two Fe^2+^/α-KG-dependent dioxygenases, FTO and ALKBH5, offering an additional layer of dynamic control (139). The dependence of these erasers on α-KG and Fe^2+^ makes them sensitive to metabolic perturbations, particularly accumulation of competing metabolites such as succinate and itaconate (139, 140). The functional readout of m^6^A is mediated by a diverse set of reader proteins (79). Cytoplasmic YTHDF proteins (YTHDF1, YTHDF2, YTHDF3) typically regulate translation and mRNA decay, with YTHDF2 promoting degradation of methylated transcripts and YTHDF1 enhancing their translation (141). Nuclear YTHDC1 and YTHDC2 influence splicing and export. The IGF2BP family (IGF2BP1, IGF2BP2, IGF2BP3) often acts to stabilize methylated transcripts, opposing the effects of YTHDF2 (142). This diversity of reader functions allows m^6^A to exert context-dependent effects on gene expression, which can vary by cell type, developmental stage, and environmental condition. While these molecular mechanisms are well established in multiple biological systems, their specific dysregulation in COPD—and the extent to which each component contributes to disease pathogenesis—remains an active area of investigation.

### 3.2 m^6^A in immune cell differentiation and effector function

In CD4^+^ T cells, METTL3 deficiency impairs Th17 differentiation (142), while FTO promotes Th17 pathogenicity (143). METTL14 facilitates Foxp3 translation and Treg function (135, 144). In macrophages, FTO promotes M1 programs, while ALKBH5 supports M2 polarization (145–147). In neutrophils, m^6^A controls transcripts such as Cxcr2, Elane, and Padi4, thereby modulating chemotaxis, elastase release, and NET formation—processes that contribute to airway tissue damage and mucus obstruction in COPD (148–152). However, most insights derive from murine or non-COPD models; direct evidence in human COPD neutrophils, macrophages, and T cells remains limited. While FTO-mediated regulation of IL-17 in human T cells has been demonstrated in autoimmune contexts, COPD-specific validation is lacking (137, 146).

### 3.3 Evidence for m^6^A dysregulation in COPD

The most compelling functional evidence comes from a single rat model study with Shenqi Tiaoshen Formula, showing METTL16-mediated MALT1 hypermethylation and NF-κB activation (82). This finding awaits independent replication in human tissues. Transcriptomic reanalysis shows altered m^6^A machinery expression (METTL16, FTO, YTHDF2) (153, 154), with FTO levels correlating with IL-17A in PBMCs (155). Other candidate transcripts whose m^6^A status may be altered in COPD include *IL-6*, *CXCL8*, *TNF*, and *NFKB1* (156). The PADI4 transcript, encoding the key NETosis enzyme PAD4, contains m^6^A sites that affect its stability, as demonstrated in neutrophil-like cell lines (157–159). However, these latter observations derive from non-COPD contexts and await confirmation in COPD-relevant systems.

### 3.4 Interplay between metabolism and m^6^A regulation: a bidirectional circuit

Metabolism and m^6^A form a bidirectional circuit (160). In the following discussion, we distinguish between established mechanisms (with direct experimental evidence across multiple systems) and hypothetical ones (supported by indirect evidence and requiring validation specifically in COPD contexts). SAM is the obligate methyl donor for m^6^A writers, produced by the methionine cycle which depends on methionine availability, folate-mediated one-carbon metabolism, and serine/glycine flux from glycolysis (161). This relationship has been demonstrated in multiple cell types and is mechanistically well-characterized. In COPD and other chronic inflammatory states, enhanced glycolysis can alter one-carbon metabolism and increase SAM levels, potentially favoring hypermethylation of pro-inflammatory transcripts. m^6^A itself modulates metabolic gene expression in multiple systems: METTL16 regulates *MAT2A* mRNA stability, affecting intracellular SAM pools (162); m^6^A modification of *PFKFB3* enhances its translation and stimulates glycolytic flux (162); m^6^A on *CPT1A* destabilizes this key regulator of fatty acid oxidation, impairing oxidative metabolism; and FTO-mediated demethylation stabilizes *PGC-1α* mRNA, promoting mitochondrial biogenesis and oxidative phosphorylation. These mechanisms have been validated in various cell types and disease models, though their specific operation in COPD requires direct investigation (163).

Beyond these established pathways, several mechanistic links between metabolism and m^6^A regulation remain hypothetical in the COPD context and await direct experimental verification. m^6^A erasers (FTO, ALKBH5) require α-KG as a co-substrate; perturbations of the tricarboxylic acid cycle during inflammation—such as truncation leading to succinate accumulation—can inhibit these α-KG-dependent dioxygenases through competitive inhibition (164). While this mechanism has been demonstrated in cancer and other inflammatory contexts, direct evidence in COPD macrophages is currently lacking. In COPD macrophages, succinate accumulation would therefore be expected to reduce demethylase activity and favor persistent m^6^A marks, but this remains speculative until directly tested. Itaconate, produced by activated macrophages from citrate via immune responsive gene 1 (IRG1), has been shown to modulate m6A modification through the OGDH-itaconate pathway, and ALKBH5 activity has been reported to regulate OGDH expression (14). However, direct evidence for itaconate-mediated inhibition of ALKBH5 in COPD macrophages is currently lacking, and this represents an important area for future investigation. These observations come primarily from studies in cancer and infectious disease models; their relevance to COPD requires direct investigation.

The effects described above can consolidate a self-reinforcing pro-inflammatory metabolic state: inflammation-driven glycolysis increases SAM and succinate, promoting m^6^A accumulation on pro-inflammatory and glycolytic transcripts while suppressing oxidative metabolism, thereby sustaining chronic inflammation. However, the extent to which this circuit operates in human COPD lung tissues remains to be directly demonstrated, and the causal hierarchy—whether metabolic changes drive epitranscriptomic alterations or vice versa—has not been established in COPD.

### 3.5 TCM and m^6^A: mechanistic evidence and therapeutic implications

Shenqi Tiaoshen Formula downregulation and reduced *MALT1* m^6^A methylation in a rat model (82). Other TCM formulas (Yupingfeng San, Bu Zhong Yi Qi Tang) show potential m^6^A-modulating activity in non-COPD contexts (165). The active compounds discussed in Section 2 have also been studied for m^6^A-modulating effects in other disease models, as summarized below. In each case, the evidence derives from non-COPD contexts and requires validation in COPD-relevant systems: baicalin inhibits METTL3 and upregulates FTO in lung cancer cells (166); astragaloside IV downregulates METTL3 in renal fibrosis models (167); berberine inhibits FTO while upregulating ALKBH5 in metabolic syndrome contexts (168); curcumin concurrently inhibit METTL3 and FTO in colitis models (169); and resveratrol upregulates ALKBH5 in neuroinflammatory paradigms, promoting resolution of inflammation (170). Beyond direct effects on m^6^A enzymes, these compounds also modulate metabolism and NETosis, positioning them to interrupt the coupled metabolic–epitranscriptomic circuit that sustains COPD inflammation. However, the m^6^A-modulating effects of these compounds require direct validation in COPD-relevant systems—including primary human airway cells, COPD patient samples, and physiologically relevant concentrations—before they can be confidently integrated into the mechanistic model of TCM action in COPD.

### 3.6 Gaps, limitations, and key experimental priorities

Most human data are correlative; the Shenqi Tiaoshen study is limited to one animal model and transcript. Effects attributed to individual compounds derive from non-COPD contexts at supraphysiological concentrations. Priorities include high-resolution epitranscriptomic mapping in human COPD, CRISPR manipulation in primary cells, and systematic evaluation of TCM in COPD-relevant human models with PK/PD profiling.

### 3.7 Therapeutic opportunities and conceptual frameworks

The integrated metabolic–epitranscriptomic model of COPD suggests multiple therapeutic strategies. Directly targeting m^6^A writers or erasers with small molecules that inhibit METTL3/METTL16 or activate FTO/ALKBH5 could decrease pathogenic methylation on pro-inflammatory transcripts. However, global modulation risks pleiotropic effects given the pervasive role of m^6^A in tissue homeostasis. Therefore, approaches that provide selective targeting (e.g., nanoparticle delivery to lung macrophages or inhaled formulations) or transient modulation during exacerbations may improve therapeutic windows. Metabolic modulation to indirectly reprogram m^6^A—by reducing glycolytic flux or normalizing TCA cycle function—could lower SAM availability and restore α-KG-dependent demethylase activity, indirectly reducing pathogenic m^6^A. This approach has the advantage of broader metabolic correction and may synergize with direct epitranscriptomic interventions. Multi-target TCM formulations and natural compounds with polypharmacology, simultaneously affecting metabolism, inflammatory signaling, and m^6^A enzymes, may be well suited to modulate the self-reinforcing circuits that sustain COPD inflammation. The Shenqi Tiaoshen Formula demonstrates that such multi-component therapies can downregulate METTL16 and reduce pathogenic m^6^A on a pro-inflammatory transcript. Rationally optimized TCM-inspired combinations, with rigorous standardization and mechanistic validation, may provide a translationally feasible path forward. However, it must be emphasized that the therapeutic opportunities outlined here are based on a conceptual framework that remains incompletely validated in human COPD. The path to clinical translation will require systematic testing in human primary cells, biomarker-rich early-phase trials, and rigorous assessment of target engagement before proceeding to large-scale efficacy studies.

## 4 NETosis and defective resolution in COPD - TCM modulation

Neutrophils are the predominant innate immune cells in COPD airways, and NETosis has emerged as a central mediator of tissue injury (171). This process intersects with immunometabolism, epitranscriptomics, and impaired resolution. Preclinical evidence suggests TCM can modulate NETosis, but COPD-specific validation is limited.

### 4.1 Mechanisms and metabolic requirements

NETosis encompasses a spectrum of regulated processes that culminate in the extrusion of chromatin decorated with neutrophil elastase (NE), myeloperoxidase (MPO), cathepsin G, and other antimicrobial proteins (172). Classical suicidal NETosis requires NOX2-generated ROS, which initiate downstream signaling cascades (173). A central enzymatic event is activation of PAD4, which catalyzes histone citrullination and promotes chromatin decondensation (174). In parallel, granular enzymes such as NE translocate to the nucleus and contribute to chromatin decondensation by cleaving histones (175). Alternative “vital” NETosis pathways allow extrusion of nuclear or mitochondrial DNA while retaining some functional capacity (176). Importantly, NETosis is metabolically demanding and intimately linked to cellular bioenergetics (177). High glycolytic flux supports rapid ATP production and, through the pentose phosphate pathway, supplies NADPH required for NOX2-driven reactive oxygen species generation. Studies using inhibitors of glycolysis (2-deoxyglucose) or the pentose phosphate pathway (dehydroepiandrosterone) suppress NET formation, demonstrating this metabolic dependency (178). Mitochondrial function, reactive oxygen species production, and calcium fluxes also shape NET formation in stimulus- and context-dependent manners. Thus, metabolic reprogramming of neutrophils—whether toward glycolysis or altered mitochondrial respiration—directly influences NETogenic capacity (179).

### 4.2 Excessive NETosis in COPD

Sputum and lung tissue from COPD patients show elevated H3Cit and NE-DNA/MPO-DNA complexes, correlating with disease severity (180). Cigarette smoke, the principal etiologic exposure in many COPD cases, directly induces NETosis via reactive oxygen species generation and PAD4 activation (181).

### 4.3 Pathogenic consequences of NETs in the COPD lung

NETs cause proteolytic tissue destruction (NE, MPO degrade elastin and inactivate antiproteases) (182). drive mucus hypersecretion via TLR4-NF-κB (183), and expose citrullinated autoantigens (184). A subset of COPD patients develops anti-citrullinated protein antibodies (185). Defective macrophage efferocytosis in COPD fails to clear NETs (186), while NET-associated elastase degrades SPMs (e.g., LXA_4_) (187). Reduced SPM levels in COPD and exogenous SPM attenuation of inflammation in models underscore resolution failure.

### 4.4 Intersections with immunometabolism and epitranscriptomic regulation

Glycolysis and the PPP are essential for NETosis (178); AMPK activation suppresses it (188). m^6^A modifications influence Padi4 mRNA stability (189, 190). Metabolic shifts (succinate, itaconate) may promote m^6^A remodeling stabilizing NET-promoting transcripts, but this framework is inferred from non-COPD contexts.

### 4.5 TCM modulation of NETosis and resolution

Preclinical evidence from non-COPD models shows TCM compounds inhibit NETosis: berberine inhibits PAD4 and NOX2 (191, 192); curcumin suppresses PAD4 (193); tetrandrine degrade pre-formed NETs (194). Antioxidant constituents including astragaloside IV, resveratrol, and flavonoids act as ROS scavengers, indirectly limiting NET release (195). Regarding protease inhibition and mitigation of NET-mediated tissue damage, baicalin inhibits neutrophil elastase activity and mitigates elastase-induced lung injury in experimental models, which could protect against NET-driven matrix degradation and emphysema progression (196). Saikosaponin D suppresses neutrophil elastase release from activated neutrophils, reducing proteolytic burden (15). Xuefu Zhuyu Decoction and Danzhi Jiangtang Capsule increased LXA_4_ levels in models of acute lung injury and diabetic nephropathy, respectively, demonstrating capacity to augment SPM biosynthesis (197–199). The Shenqi Tiaoshen Formula has demonstrated reduced NE activity, increased LXA_4_ in BALF, and improved NET clearance by alveolar macrophages in COPD rat models (82). Yupingfeng San, by increasing secretory IgA and reducing airway inflammation, may facilitate opsonization and macrophage clearance of NETs, representing an indirect but potentially clinically relevant mechanism (200).

### 4.6 Gaps and translational research

Most studies use supraphysiological concentrations and non-COPD models. Global NETosis suppression may impair host defense—a concern highlighted by increased infection risk with broad-spectrum immunomodulators. Thus, strategies must balance antimicrobial preservation with tissue protection. Targeted delivery or temporally limited interventions may mitigate risks (198). Priorities include standardized preparations, PK/PD profiling, ex vivo assays using patient-derived cells, and biomarker-rich early trials (201).

## 5 Connecting the dimensions — How TCM intercepts a self-perpetuating network in COPD

The preceding sections have outlined three interdependent pathobiological processes in COPD: metabolic reprogramming, m^6^A RNA methylation, and NET formation coupled with impaired resolution. These processes do not operate in isolation; rather, they form a tightly interlocked, self-sustaining network in which perturbation of any single node propagates and amplifies dysfunction across the system. Recognizing this integrative architecture clarifies why single-target interventions frequently fail to produce durable clinical benefit in established COPD, and why multi-targeted therapeutics—including rationally formulated TCM combinations—may be uniquely suited to interrupt the vicious cycle. However, it is important to emphasize that the network model presented here is a conceptual synthesis derived from evidence across multiple model systems; the degree of validation for individual connections varies considerably, and many links remain speculative until directly tested in human COPD tissues.

### 5.1 An integrated pathogenic circuit

Chronic exposures initiate innate immune activation, inducing metabolic reprogramming (aerobic glycolysis, enhanced PPP) that supplies ATP and NADPH for ROS and NETosis (202). Regulatory cells (e.g., Tregs and ILC2s) show impaired FAO and mitochondrial biogenesis (203). Metabolites (SAM, succinate, itaconate) bias the epitranscriptome toward hypermethylation (204). m^6^A stabilizes MALT1, PFKFB3 and destabilizes CPT1A, reinforcing glycolysis and inflammation (205). Enhanced glycolysis and NADPH favor NETosis (206), causing tissue damage, autoantigen exposure, and SPM degradation (207). Failure of alveolar macrophages clearance prolongs inflammation, feeding back to metabolic stress (208). Collectively, these interconnections create a resilient, self-perpetuating inflammatory state that is difficult to extinguish with single-mechanism therapies. The conceptual framework presented here is supported by evidence from multiple experimental systems, but the causal hierarchy among these processes—and the relative contribution of each node to disease persistence—remains incompletely defined in human COPD.

### 5.2 Points of interception and TCM actions

**Figure 2:**
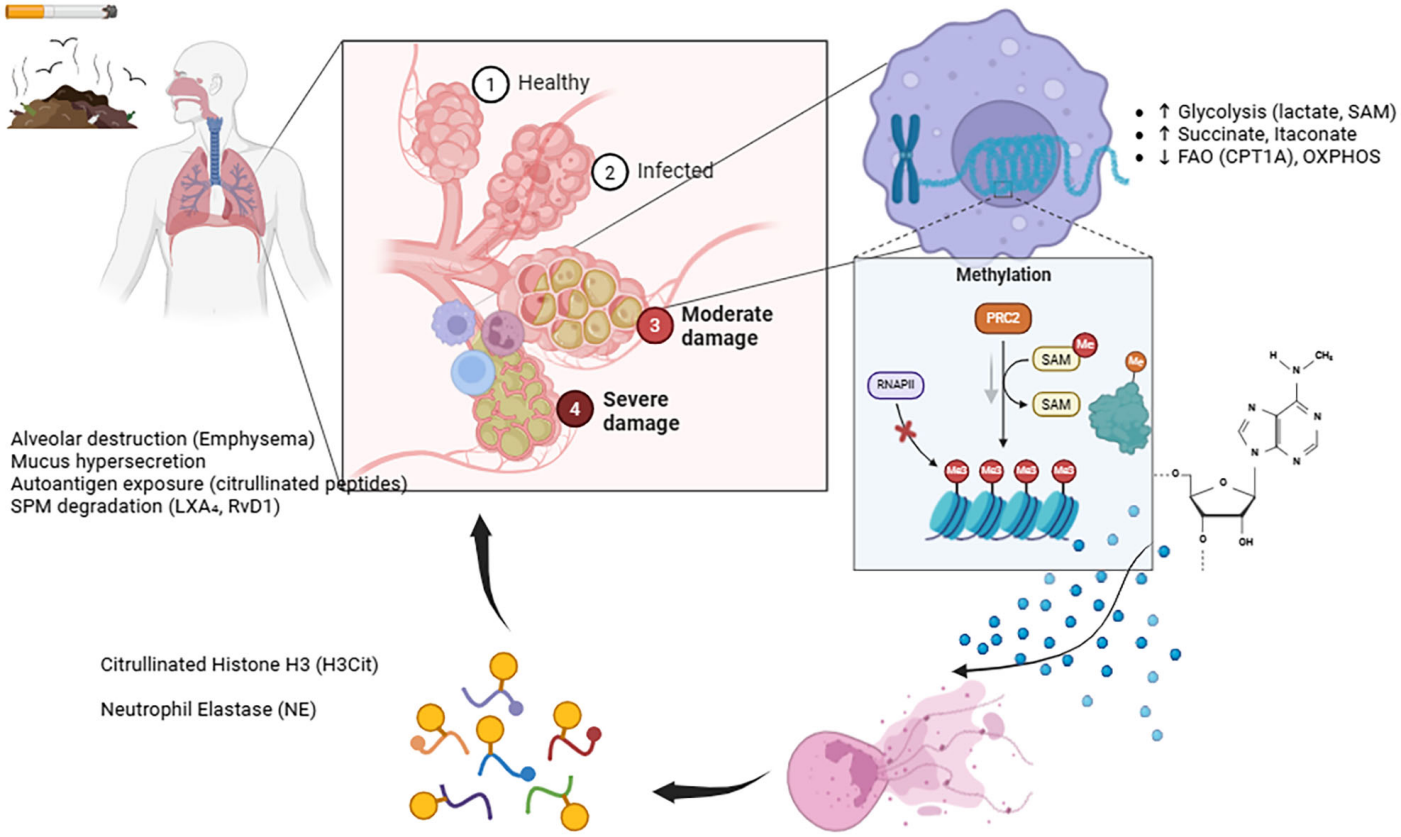
The m^6^A-centered pathogenic network integrating immunometabolism, epitranscriptomics, and NETosis in COPD. Chronic exposures induce immunometabolic reprogramming (increased glycolysis, SAM/succinate accumulation, impaired FAO), which shifts m^6^A balance toward hypermethylation via upregulation of METTL16 and downregulation of FTO/ALKBH5. Hypermethylation of MALT1, PADI4, and PFKFB3 stabilizes these transcripts, enhancing NF-κB signaling, NETosis, and glycolysis, while destabilization of CPT1A impairs FAO. NETs cause alveolar destruction, mucus hypersecretion, autoantigen exposure, and SPM degradation, and feedback to perpetuate metabolic and epitranscriptomic dysregulation. TCM interventions (Shenqi Tiaoshen Formula, berberine, baicalin, astragaloside IV, curcumin) intercept this network at multiple nodes, as indicated. Abbreviations are provided in the manuscript.

TCM can impact multiple nodes simultaneously (209). Metabolic normalization via AMPK activation (berberine, astragaloside IV) shifts metabolism toward FAO, lowers NADPH and SAM, and restores m^6^A balance (210). PPARγ induction promotes FAO, SPM biosynthesis, and NET clearance (91). Direct epitranscriptomic modulation: Shenqi Tiaoshen Formula downregulates METTL16 (82); other compounds, including baicalin and astragaloside IV, have been observed to affect METTL3 expression in non-COPD contexts, offering potential translational leads (211). Natural products such as baicalin and resveratrol have also been reported to upregulate the erasers FTO or ALKBH5 in other disease models (23). Restoring eraser activity can counter succinate-mediated inhibition and reduce pathogenic m^6^A marks, decreasing PAD4 and PFKFB3 expression and thereby dampening NETogenic and glycolytic phenotypes (139)—though this sequence remains speculative in COPD (**Figure 2**).

### 5.3 Principles for TCM formula design and synergistic combinations

Given the networked pathology, TCM formulas should be rationally composed to target complementary nodes rather than rely on empirical tradition alone. Based on the network medicine framework and the mechanistic insights reviewed above, we propose the following design principles.

Multi-node engagement is preferable, meaning that formulas should include components that modulate metabolism (AMPK, PPARγ), epitranscriptomic enzymes (writers/erasers), NET triggers (PAD4, ROS), and resolution (SPM biosynthesis, efferocytosis). Temporal sequencing may also be beneficial, with formulations or dosing regimens that initially suppress acute NETosis and proteolysis, then promote metabolic reprogramming and resolution to consolidate repair. Dose and specificity should be carefully considered to achieve lung-relevant target engagement with minimal systemic suppression of host defense; inhaled or otherwise lung-targeted delivery may increase the therapeutic index. Finally, rigorous standardization with active constituent quantification and reproducible extraction methods is essential for mechanistic studies and clinical trials.

### 5.4 Biomarker development and patient stratification

Biomarker-driven stratification is critical. Composite indices combining metabolic (succinate, lactate, SAM/SAH), epitranscriptomic (METTL16/METTL3, FTO, ALKBH5), and NET (H3Cit, NE-DNA) measures can define endotypes (212). Immunometabolic-epitranscriptomic endotypes—for example, high NET/high m^6^A/high glycolysis versus low NET/high autoantibody presence—can be classified to predict responsiveness to specific TCM combinations. Pharmacodynamic readouts using reduction in sputum H3Cit, normalization of SAM/α-KG ratios, restored SPM levels, and improved macrophage efferocytosis ex vivo can serve as early indicators of biological activity prior to clinical outcomes (213).

As noted in subsequent sections, many of the biomarker assays proposed above remain research tools rather than clinically validated tests. MeRIP-qPCR for m^6^A on specific transcripts requires specialized expertise and is not standardized across laboratories. Spatial metabolomics requires specialized instrumentation not available in most clinical settings. Even the more accessible assays—H3Cit ELISA and LC-MS/MS for SPMs—require standardization of pre-analytical handling, reference range establishment, and cross-laboratory validation before they can be deployed in multicenter trials or clinical practice. A pragmatic path forward involves using research-grade assays in early-phase mechanistic studies with centralized laboratory analysis, progressively transferring and standardizing the most promising assays into clinical laboratory settings, and validating candidate biomarkers in prospective cohorts before incorporating them into pivotal trials.

### 5.5 Clinical translation and trial design

The translation of these findings into clinical practice would benefit from a staged, mechanism-driven approach. Preclinical co-validation using human primary cell models (neutrophils, macrophages, airway epithelial cells), organoids, and precision-cut lung slices can demonstrate target engagement, metabolic/epitranscriptomic modulation, reduced NETosis, and enhanced resolution, employing inhalation delivery prototypes where feasible (213).

Early human proof-of-biology trials should be small, biomarker-rich studies in stratified COPD cohorts (selected by elevated NET biomarkers or metabolic/epitranscriptomic signatures) to demonstrate safety, target engagement, and relevant pharmacodynamic effects (reduced NET burden, restored SPM levels, improved sputum rheology) (214). These trials should incorporate rigorous pharmacokinetic sampling to confirm that active constituents reach the lung at concentrations sufficient for target engagement—a critical consideration given the bioavailability challenges discussed in Section 2.6.

Adaptive efficacy trials should enroll biomarker-defined subgroups into adaptive randomized controlled trials to test clinical endpoints (exacerbation frequency, lung function decline, symptoms, quality of life) while continuing mechanistic sampling to refine endotype-response relationships (185). Safety vigilance is mandatory because interventions touch pathways involved in host defense and global gene regulation (m^6^A); rigorous monitoring for infection risk, unintended immunosuppression, and off-target epigenetic effects will be required.

A critical gap in the proposed biomarker strategy is the limited clinical readiness of many assays. A pragmatic staged approach may address this limitation. In early-phase mechanism studies, research-grade assays with rigorous quality control should be used, run in centralized, specialized laboratories, focusing on demonstrating target engagement and biological activity (assays in this category include MeRIP-qPCR for m^6^A on specific transcripts, spatial metabolomics, and single-cell m^6^A sequencing). For promising biomarkers, assays should be transferred to clinical laboratories with standardized protocols, quality control materials, and reference ranges, partnering with diagnostic companies for commercial assay development (assays in this category include H3Cit ELISA, NE–DNA complexes, and LC-MS/MS for SPMs). For biomarkers that consistently predict clinical outcomes, implementation in clinical practice with established reference ranges and clinical decision-making algorithms would represent the final step.

### 5.6 Challenges and research priorities

Despite the conceptual appeal of the integrated network model and the multi-target potential of TCM, several formidable challenges must be addressed. The heterogeneity of COPD pathology means that no single intervention is likely to benefit all patients; biomarker-driven stratification is therefore essential. The potential trade-offs between suppressing NETosis and preserving antimicrobial defense require careful dose optimization and possibly intermittent or targeted delivery. The pleiotropic roles of m^6^A across tissues raise concerns about off-target effects of global epitranscriptomic modulation. The complexity of standardizing multi-component formulas presents regulatory and manufacturing challenges (215). A particularly important challenge is achieving therapeutic concentrations of TCM compounds in lung tissue, given the poor oral bioavailability of many active constituents (Section 2.6) (216).

High-resolution mapping using single-cell multi-omic profiling (transcriptome, m^6^A epitranscriptome, metabolome, lipidome) in COPD airways should be pursued to delineate cell type-specific circuits (217). Target validation through causal perturbation studies (CRISPR, pharmacologic) linking specific m^6^A modifications and metabolic shifts to NETosis and clinical phenotypes represents another priority. Pharmacokinetic/pharmacodynamic and delivery development through lung-targeted formulations and robust PK/PD models for TCM constituents are also needed (218); Formulation strategies including inhaled dry powder formulations, liposomal encapsulation, polymeric nanoparticles, and ligand-conjugated carriers that target alveolar macrophages (e.g., mannose- or folate-conjugated nanoparticles) warrant investigation, as these approaches can achieve higher local concentrations with reduced systemic exposure, potentially overcoming bioavailability limitations. Finally, safety and immunologic balance should be addressed through preclinical infection models and careful clinical monitoring to detect compromised host defense, with clinical studies incorporating lung tissue sampling (e.g., sputum, BAL, exhaled breath condensate) to confirm target engagement (219).

## 6 Emerging technologies to advance mechanism-driven TCM research in COPD

Translating TCM into precision therapies for COPD requires technologies that can resolve biological complexity at cellular, molecular, spatial, and temporal scales (220). The network linking immunometabolism, m^6^A RNA methylation, and NETosis is multi-layered and highly cell type-specific (133). Conventional bulk assays are insufficient to map the heterogeneity, cellular interactions, and local microenvironments that determine therapeutic response. Recent advances in single-cell and spatial technologies, functional genomics, and multi-omics integration offer powerful tools to address these challenges (221). However, it should be noted that most of these technologies remain in the research domain; their translation to clinical trial settings—particularly for TCM research—requires substantial assay development, standardization, and validation before they can be deployed as decision-making tools in multicenter studies or clinical practice. The following discussion therefore focuses on research applications and the translational pathway toward clinical implementation.

### 6.1 Single-cell m^6^A sequencing: resolving epitranscriptomic heterogeneity

Single-cell m^6^A sequencing methods such as scDART-seq (single-cell Differential Analysis of m^6^A with Reverse Transcription sequencing) and m^6^A-CITE-seq enable high-resolution mapping of methylation at the level of individual cells (222). These technologies combine the throughput of single-cell RNA-seq with antibody-based capture of m^6^A-modified RNA, allowing simultaneous measurement of transcript abundance and methylation status (223). Applying these to bronchoalveolar lavage fluid and dissociated lung tissue from COPD patients before and after TCM treatment can identify which cell clusters harbor pathogenic m^6^A remodeling—for example, macrophage subsets with METTL16 upregulation or neutrophil subclusters with hypermethylated *PADI4* (137). Pairing m^6^A maps with single-cell transcriptomes and surface immunophenotyping allows tracking of dynamic epitranscriptomic changes during exacerbations and in response to therapy (224).

It is important to recognize, however, that single-cell m^6^A sequencing technologies are still evolving. Current methods have limited throughput compared to standard single-cell RNA-seq, and the coverage of m^6^A sites per cell remains sparse. Furthermore, these techniques have been applied primarily to cell lines or mouse tissues; their application to human lung samples—particularly archived clinical specimens—faces challenges related to tissue dissociation, cell viability, and RNA integrity. For TCM research specifically, these methods have not yet been applied to evaluate herbal formula effects in human COPD samples. Priority areas include optimization of protocols for clinical lung samples (bronchial biopsies, BAL cells, sputum cells), development of multiplexing strategies to increase throughput and reduce costs, integration with single-cell metabolomics to directly link epitranscriptomic changes to metabolic states, and application to TCM intervention studies in well-phenotyped COPD cohorts to identify cell type-specific responses.

### 6.2 Spatial metabolomics: mapping the metabolic landscape

Spatial metabolomics using matrix-assisted laser desorption/ionization mass spectrometry imaging can map small molecules (SAM, succinate, itaconate, lactate, citrate) with micrometer resolution on tissue sections (225). Combined with spatial transcriptomics platforms (e.g., Visium, Xenium, MERFISH), this approach can identify microenvironments with high SAM to SAH ratios, succinate hotspots, and co-localized m^6^A enzyme expression, and overlay these with NET biomarkers and SPM levels to visualize intersections of pathology (226). Isotopically labeled TCM constituents can also be used to map tissue distribution and accumulation of active compounds in inflamed regions, providing crucial pharmacokinetic information (227).

While spatial metabolomics offers unprecedented resolution, several barriers to clinical application exist. The instrumentation (MALDI-MSI) requires specialized facilities and expertise, and sample preparation protocols are not standardized across laboratories. Data analysis and interpretation require sophisticated bioinformatics pipelines. For TCM research, the application of spatial metabolomics to evaluate herbal formula effects in human lung tissues has not yet been reported. A pragmatic pathway forward would involve first applying these technologies to preclinical models (COPD mouse models treated with TCM compounds) to establish feasibility and identify candidate metabolic hotspots, then validating findings using targeted metabolomics in human biospecimens (BAL, sputum, plasma), and gradually translating spatial approaches to human tissues once protocols are optimized and standardized.

### 6.3 Functional genomics: identifying causal nodes in the network

Functional genomics can reveal which m^6^A regulators and metabolic enzymes are necessary for TCM efficacy (228). Pooled CRISPR knockout libraries targeting m^6^A writers, erasers, readers, metabolic enzymes (*MAT2A*, succinate dehydrogenase subunits, *IRG1, CPT1A*), PAD enzymes, and SPM biosynthetic genes can be screened in COPD-relevant human cells (primary alveolar macrophages, iPSC-derived neutrophils, airway epithelial organoids) treated with standardized TCM extracts (229). Readouts may include NF-κB activity, NETosis metrics (citrullinated histone H3 ELISA or imaging cytometry), metabolic flux (extracellular acidification rate, oxygen consumption rate), and cell viability under cigarette smoke extract exposure (176). Orthogonal validation with single-gene perturbations and rescue experiments confirms causal links. Emerging CRISPR activation and interference platforms allow fine-tuned modulation of gene expression without complete knockout, which may better model the effects of pharmacological interventions (230).

For CRISPR screens to be informative in the context of TCM research, several design considerations are critical. First, the choice of cell model matters: primary human cells—particularly alveolar macrophages and neutrophils from COPD patients—are preferable to immortalized cell lines, though they present technical challenges for CRISPR delivery and culture. Second, the concentration of TCM extracts used in screens should be physiologically relevant (see Section 2.6), with parallel pharmacokinetic studies to confirm that active constituents reach the target cells at effective concentrations. Third, screens should be performed in the presence of relevant stimuli (cigarette smoke extract, bacterial LPS) to model the inflammatory COPD environment. Fourth, hits from pooled screens require validation in patient-derived cells and, ultimately, in clinical samples to confirm relevance to human disease.

### 6.4 Biomarker panels for clinical translation: from research tools to clinical assays

For clinical translation, candidate biomarker panels can be developed using minimally invasive sampling (231). Epitranscriptomic markers can be measured by targeted MeRIP-qPCR for specific transcripts (*MALT1, PADI4, PFKFB3, CPT1A*) in sputum cells or circulating extracellular vesicles (232). Metabolic markers include plasma or sputum SAM to SAH ratio (measured by liquid chromatography-mass spectrometry), succinate, lactate, and itaconate (233). NET and resolution markers include sputum or plasma citrullinated histone H3, neutrophil elastase–DNA complexes, myeloperoxidase–DNA, and SPM quantification by liquid chromatography-tandem mass spectrometry (217). Implementing these in early-phase clinical studies of standardized TCM interventions demonstrates target engagement (e.g., reduced sputum MALT1 m^6^A, lowered citrullinated histone H3) before large efficacy trials. Composite indices integrating these markers define biomarker-driven endotypes that can guide patient selection in future trials (234).

As discussed in Sections 5.4 and 5.5, a critical bottleneck is the limited clinical readiness of many candidate biomarker assays. While research-grade versions of these assays exist, their translation to Clinical Laboratory Improvement Amendments (CLIA)-certified or equivalent clinical laboratory settings requires substantial investment in standardization. Key steps include establishing standard operating procedures for sample collection, processing, storage, and analysis; developing quality control materials with known concentrations of analytes; defining reference ranges in healthy controls and COPD patients; validating analytical performance (precision, accuracy, linearity, limits of detection); conducting multi-center reproducibility studies; and obtaining regulatory approval where required. For TCM research specifically, a pragmatic approach would prioritize the most accessible and well-validated assays for early-phase trials (e.g., H3Cit ELISA, sputum lactate), while reserving more specialized assays (e.g., MeRIP-qPCR, LC-MS/MS for SPMs) for centralized analysis in mechanism-focused substudies.

### 6.5 Computational integration and network modeling

Computational models that integrate multi-omic data into actionable hypotheses are essential for realizing the potential of TCM in COPD (235). Network pharmacology approaches link known compound–protein interactions (from databases such as TCMSP, STITCH, ChEMBL) with disease networks derived from human multi-omics data, enabling prediction of which TCM formulas are most likely to modulate specific pathogenic circuits (236). Causal inference and graphical models (Bayesian networks, causal mediation analysis) can distinguish direct from indirect effects of treatments in observational and interventional studies. Machine learning for endotype discovery clusters integrated single-cell epitranscriptomic, metabolomic, and proteomic data to define clinically meaningful endotypes and predict therapy response.

The application of network modeling to TCM is particularly promising given the multi-component nature of herbal formulas. Rather than modeling individual compounds in isolation, network approaches can capture the combined effects of formula constituents on disease modules. Key challenges include the incompleteness of compound–target databases for TCM constituents, particularly for metabolites and degradation products; the difficulty of modeling indirect effects mediated through the gut microbiome; the need for time-series data to capture dynamic responses to TCM interventions; and the challenge of validating computational predictions experimentally.

### 6.6 A proposed translational pipeline integrating these technologies

Preclinical discovery and standardization would involve chemical characterization of TCM formulas using high-performance liquid chromatography-mass spectrometry; *in vitro* ADME (absorption, distribution, metabolism, excretion) profiling; and mechanistic screens in human primary cells with single-cell readouts. Proof-of-biology human studies would involve small (20–50 patients per group), biomarker-driven trials demonstrating target engagement (changes in m^6^A marks, NET burden, SPM levels) and safety, using adaptive designs to drop ineffective dose levels early. Adaptive efficacy trials would involve larger studies (100–300 patients per group) enriching for biomarker-defined responders, incorporating mechanistic endpoints alongside clinical outcomes (exacerbation rate, lung function, quality of life), using response-adaptive randomization to assign more patients to promising regimens. Implementation and precision care would involve integration of validated biomarker panels into clinical algorithms to guide TCM therapy selection in routine practice, supported by prospective validation studies. Rigorous standardization of botanical sourcing, extraction, and batch testing is mandatory to meet regulatory standards from agencies such as the NMPA (China), FDA (USA), and EMA. Because NETosis and m^6^A regulate host defense, long-term safety and infection surveillance are integral parts of clinical development. Interdisciplinary collaboration among clinicians, immunologists, systems biologists, chemists, and TCM experts is essential to realize the potential of these emerging technologies. Finally, it must be emphasized that the pipeline described here is a roadmap, not a guaranteed pathway; each step will require iterative refinement based on emerging data, and the timeline for clinical translation will depend on the pace of assay development, validation, and regulatory engagement.

## 7 Concluding remarks

COPD remains a major global health challenge because its underlying inflammation is driven by complex, self-reinforcing networks rather than single molecular abnormalities. This review has integrated three emerging fields—immunometabolism, m^6^A RNA methylation, and NETosis with inflammation resolution—into a unified narrative. We have shown that TCM formulas and active compounds can modulate each of these processes, either directly or through cross-regulatory interactions. However, it is critical to emphasize that the vast majority of evidence derives from animal models, *in vitro* systems with compound concentrations that often exceed physiologically achievable levels, and non-COPD disease contexts. Direct evidence in human COPD tissues and primary cells remains limited. The discovery that the Shenqi Tiaoshen Formula reduces METTL16-mediated m^6^A methylation of *MALT1* provides a paradigm shift, demonstrating that TCM can act at the level of RNA modification in an animal model; this finding awaits replication in human systems. The multi-target actions of berberine, baicalin, astragaloside IV, curcumin, and resveratrol exemplify how single TCM-derived compounds can simultaneously affect metabolism, RNA methylation, and NETosis. Key future priorities include high-resolution mapping of m^6^A landscapes in human COPD lungs across disease stages and cell types; causal validation of the metabolic–epitranscriptomic–NET axis in human primary cells using CRISPR and pharmacological tools; rigorous standardization and PK/PD characterization of TCM formulas; biomarker-driven clinical trials that stratify patients by metabolic, epitranscriptomic, and NET signatures; and development of lung-targeted delivery systems (inhaled nanoparticles, dry powder formulations).

By embracing the interconnected nature of these processes and systematically addressing the evidentiary gaps identified above, the TCM field may move beyond descriptive pharmacology and contribute to a new era of network-based, holistic therapy for COPD. Achieving this goal, however, will require sustained investment in mechanistic research, rigorous clinical validation, and interdisciplinary collaboration.
